# Universal diffusion-limited injection and the hook effect in organic thin-film transistors

**DOI:** 10.1038/srep29811

**Published:** 2016-07-21

**Authors:** Chuan Liu, Gunel Huseynova, Yong Xu, Dang Xuan Long, Won-Tae Park, Xuying Liu, Takeo Minari, Yong-Young Noh

**Affiliations:** 1 State Key Laboratory of Optoelectronic Materials and Technologies, Guangdong Province Key Laboratory of Display Material and Technology, SYSU-CMU Shunde International Joint Research Institute, School of Electronics and Information Engineering, Sun Yat-sen University, Guangzhou 510275, People’s Republic of China; 2 Department of Energy and Materials Engineering, Dongguk University, 30 Pildong-ro, 1 gil, Jung-gu, Seoul 04620, Republic of Korea; 3 International Center for Materials Nanoarchitectonics (WPI-MANA), National Institute for Materials Science (NIMS), Tsukuba, Ibaraki 305-0044, Japan

## Abstract

The general form of interfacial contact resistance was derived for organic thin-film transistors (OTFTs) covering various injection mechanisms. Devices with a broad range of materials for contacts, semiconductors, and dielectrics were investigated and the charge injections in staggered OTFTs was found to universally follow the proposed form in the diffusion-limited case, which is signified by the mobility-dependent injection at the metal-semiconductor interfaces. Hence, real ohmic contact can hardly ever be achieved in OTFTs with low carrier concentrations and mobility, and the injection mechanisms include thermionic emission, diffusion, and surface recombination. The non-ohmic injection in OTFTs is manifested by the generally observed hook shape of the output conductance as a function of the drain field. The combined theoretical and experimental results show that interfacial contact resistance generally decreases with carrier mobility, and the injection current is probably determined by the surface recombination rate, which can be promoted by bulk-doping, contact modifications with charge injection layers and dopant layers, and dielectric engineering with high-*k* dielectric materials.

The charge injection at the metal–semiconductor interface is one of the main limiting factors of many non-silicon-based electronic devices, especially those with organic semiconductors^1^. The energy barriers and/or interface states at the heterojunction contacts considerably consume voltage drop^2^,^3^, generate Joule heating^4^, and decrease injection efficiency. Especially, a large contact resistance seriously suppresses the performance of thin-film transistors (TFTs) used for the back panel of displays^5^, sensors, memories, and other facile electronics, which call for a low driving voltage and a high on–off ratio^6^. Such limitations raise the demand for ohmic-contact injection^7^,^8^, which has not been achieved in most organic TFTs (OTFTs), or organic field-effect transistors (OFETs). To solve the problem, we need to understand what procedure carriers experience at the injection, as well as how contact resistance is determined by the materials, interfaces, device, and operational conditions.

Injection barriers generally exist at the contacts of OTFTs and are caused by mismatched work functions of metals with respect to the injection levels in organic semiconductors^9^, or interfacial states with charge-transfer and dipole moments^1^,^10^. The injection barrier leads to a depletion region^11^ where carriers are depleted and need to diffuse through the region after a number of collisions. The depletion width (*W*_d_) can be estimated by knowing the effective barrier height (~0.5 eV), band-gap (~2 eV), and Fermi-level of organic semiconductors (OSCs) (near the mid-gap). Using common parameters of intrinsic OSCs in the literature, *W*_d_ is estimated to be over 1 μm in OTFTs^12^. This value is much larger than the mean free path (*l*_free_) of a carrier in OSCs that is only at the order of intermolecular distance (several Å to nm) owing to structural disorders and localized polarons^13^,^14^. Moreover, the estimated *W*_d_ is much larger than the usual thickness of active layers (10 nm < *t* < 100 nm), pointing to the possibility of diffusion-limited injection. However, there is still a lack of direct evidence in OTFTs that charge injection is diffusion-limited, as well as a need for quantitative studies to understand how this limitation affects charge injection and contact resistance in OTFTs.

In this work, we analyzed different injection mechanisms and derived a general form of interfacial contact resistance (*R*_*c*,int_), which is expressed as a function of the effective injection barrier, carrier mobility, and drain voltage in staggered OTFTs. We propose that, in OTFTs with non-ohmic injection, a hook shape would appear in conductance-drain voltage (*G*-*V*_d_) relations, and we experimentally observed such an effect in OTFTs made with a broad range of materials. The hook effect indicates that as the drain field increases, OTFTs experience a transition from the injection-limited to the field-effect transport regime. By analyzing various OTFTs with our developed tool, we reveal that the interfacial contact resistance (readily excluding the bulk resistance) is closely related to carrier mobility and the charge injection in OTFTs is universally diffusion-limited. Importantly, the values of *R*_*c*,int_ followed our proposed general form in the case of surface recombination process, i.e., carriers recombine with the image charges at the metal-semiconductor interface in OTFTs. Thus, the semiconductor mobility plays a critical role in the injection, probably by determining the surface recombination velocity and rate.

## Results

### Materials and structures of OTFTs

**Figure 1:**
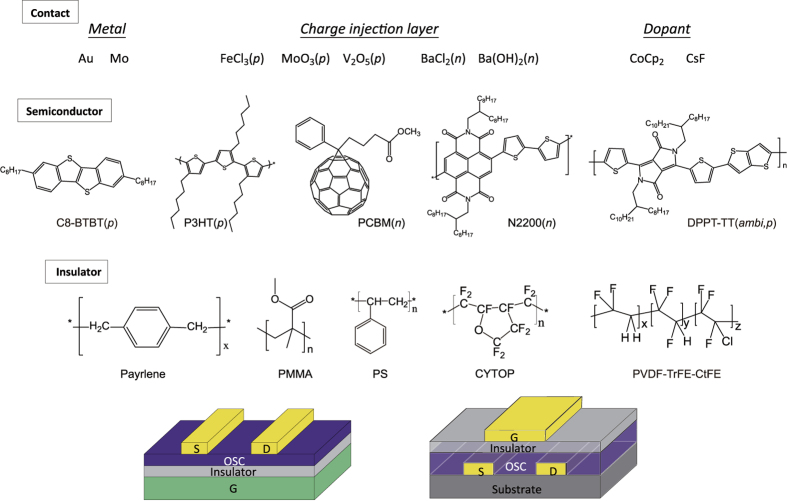
Materials for contact, semiconductors, and dielectric layers, and device structures used in this study.

To investigate the contact properties of OTFTs, we fabricated a wide range of OTFTs covering a variety of materials. The basic structures are bottom-gate/top-contact (BG/TC) and top-gate/bottom-contact (TG/BC) with gold source and drain electrodes, unless stated otherwise (see Fig. 1). For organic semiconductors, we used: (1) *p*-type small molecule dioctylbenzothienobenzothiophene (C8-BTBT) and polymer poly(3-hexylthiophene) (P3HT); (2) *n*-type small molecule [6,6]-phenyl-C61-butyric acid methyl ester (PCBM, small molecule) and polymer poly [[N,N′-bis(2-octyldodecyl)-1,4,5,8-naphthalene-dicarboximide-2,6-diyl]-alt-5,5′-(2,2′-bithiophene)] [P(NDI2OD-T2) or N2200]; and (3) ambipolar poly[[2,5-bis(2-octyldodecyl)-2,3,5,6-tetrahydro-3,6-dioxopyrrolo[3,4-c]pyrrole-1,4-diyl]-alt-[[2,2′-(2,5-thiophene) bis-thieno(3,2-b)thiophene]-5,5′-diyl]] (DPPT-TT). The materials include the commonly used thieno-thiophene-based, thiophene-based, C_60_-based, perylene-based, and diketopyrrolopyrrole (DPP)-based organic semiconductors. To modify the contacts, different types of contact engineering methods and materials were used, including selectively inserting a doping layer (FeCl_3_), inserting a charge injection layer [MoO_3_, V_2_O_5_, BaCl_2_, Ba(OH)_2_], and doping the bulk semiconductor with dopants (CoCp_2_, CsF). For the dielectrics, we selected commonly used low-*k* polymer dielectrics [parylene, poly(methyl methacrylate) (PMMA), polystyrene (PS)], and a high-*k* dielectric poly(vinylidene fluoride-ter-trifluoroethylene-ter-chlorotrifluoroethylene) [P(VDF-TrFE-CtFE)]. The semiconducting and dielectric layers of the devices were all solution-processed by spin coating, except the device with the C8-BTBT layer, which was vacuum evaporated, and the device with the parylene layer, which was chemical-vapor deposited.

### Interfacial contact resistance in OTFTs

**Figure 2:**
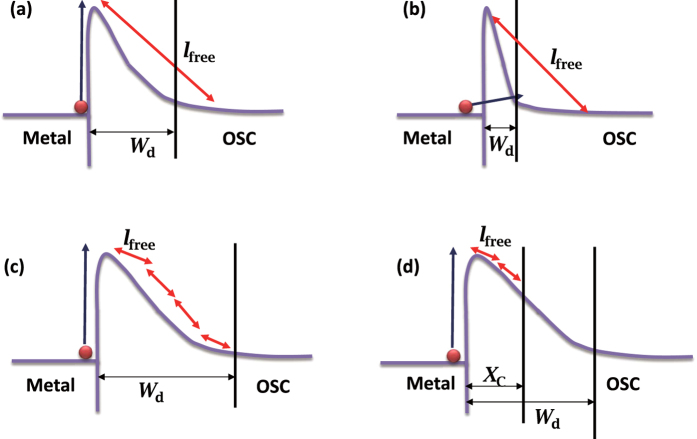
Schematic representations of charge injection mechanisms (take electron injections for an example): (**a**) thermionic emission; (**b**) tunneling; (**c**) thermionic diffusion; and (**d**) thermionic emission with surface recombination. The injections *via* thermal activation or tunneling are indicated with dark blue arrows and the mean free paths are indicated with red arrows. The characteristic lengths mentioned in the main text are also illustrated.

The injection barriers cause the injection current to increase non-linearly with the applied voltage *V*_a_ because injections may occur with the mechanisms illustrated in Fig. 2. When depletion width *W*_d_ is smaller than the mean free path *l*_free_, only thermionic emission (denoted as “e”) or tunneling processes (denoted as “t”) dominate (Fig. 2a,b) and the current follows^15^:


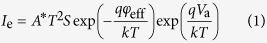



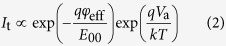


Here A* is the Richardson constant, *T* is the temperature, *k* is the Boltzmann constant, *S* is the injection area, *φ*_eff_ is the effective Schottky barrier height that takes into account the density of states distributions^16^, *E*_00_ is related to doping concentrations in semiconductors, and *V*_a_ is the applied voltage across the metal–semiconductor junction, which is assumed to follow drain voltage *V*_d_ in OTFT as *V*_a_ ∝ (*V*_d_)^α^. The image-force-induced lowering of the injection barrier (Schottky effect) is not considered here for simplicity and the forms of *R*_c,int_ are:


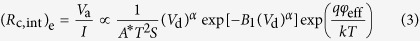



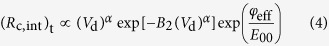


Here the parameters *B* are the constant of *V*_d_ (this also applies to the following equations).

However, when depletion width *W*_d_ is much larger than the mean free path *l*_free_, which is usually the case in OTFTs, carriers experience many collisions across the depletion region (Fig. 2c) and the injection becomes diffusion-limited. The thermionic emission–diffusion model (denoted as “ed”) predicts the injection current as^15^:


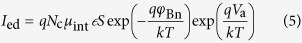


Assuming that the interfacial electric field 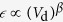 and the interfacial mobility follows the channel mobility *μ*_int_ ∝ *μ*^*γ*^, *R*_c,int_ is:


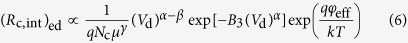


Here *q* is the elementary charge and *N*_c_ is the effective density of states in the conduction band. Especially, Emtage *et al.* showed that, at the metal–organic interface, carriers recombine with their image charges when thermal energy *kT* reaches the carrier–image binding energy^17^,^18^, and the recombination can occur with unipolar carriers. The critical distance for the recombination *x*_C_ for common OSCs (~5 nm)^18^ is larger than or comparable with *l*_free_ (several Å to a few nm) (Fig. 2d). The recombination current is opposite to the thermionic injected current and Scott *et al.* calculated the detailed balance between the two to derive the net injection current^18^:


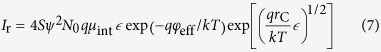


Here the subscript “r” denotes surface recombination, *ψ* is a function slowly varying with electric field *ϵ*, *N*_0_ is the total hopping sites in the organic semiconductor (similar to the above *N*_c_ in inorganic semiconductors), and *r*_C_ is the Coulomb radius (*r*_C_ ~ 4*x*_C_). The mobility appears because it determines the surface recombination velocity near the interface. This form was supported by experiments in a metal–organic semiconductor diode by Shen *et al.*^19^ and so *R*_c,int_ should follow:


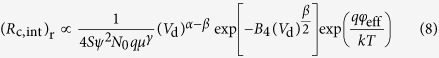


From the above models, we can summarize Eqs 2, 4, 6, and 8 to give a general form as:


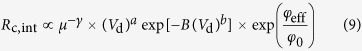


Here *φ*_0_ ideally equals 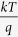 except in the tunneling model where *φ*_0_ = *E*_00_, and the parameter *B* generally decreases with thermal energy 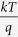. In the non-diffusion-limited cases, including thermionic emission and the tunneling models, γ = 0 and *a* = *b*; in the diffusion-limited case, including thermionic emission-diffusion and the surface recombination models, γ > 0 and usually *a *≠ *b*. We expect a diffusion-limited injection in OTFTs because *l*_free_ << *W*_d_ and it is probably accompanied by a surface recombination process as *l*_free_ ≤ *x*_C_.

### Extraction of interfacial contact resistance

In the following we will examine if *R*_c,int_ in OTFTs generally follows Eq. 9, whether charge injection in OTFTs is diffusion-limited, and, if so, to what extent the charge injection is limited by diffusion. To extract *R*_c,int_, we have derived a series of functions using the *I*_d_–*V*_d_ relations as listed in Table 1, because conventional extraction methods (e.g., transfer-length method, TLM)^20^ are not applicable as they cannot exclude the bulk injection resistance (*R*_c,bulk_). Among these functions, the *G*-function has been introduced^21^ and other functions are derived in the supplementary information (SI, part 1). The key point is that as *V*_d_ increases, *R*_c,int_ will dramatically decrease according to Eq. 9, but *R*_c,bulk_ and *R*_ch_ will gradually increase^21^; then, the conductance of the OTFT increases first and then starts to decrease at a critical drain voltage *V*_d_’. Thus, for both static output conductance 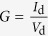 and dynamic output conductance 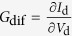, a hook shape appears near *V*_d_’ in the *G*–*V*_d_ and *G*_dif _–*V*_d_ relation (referred to as the “hook effect” in the following).

**Figure 3:**
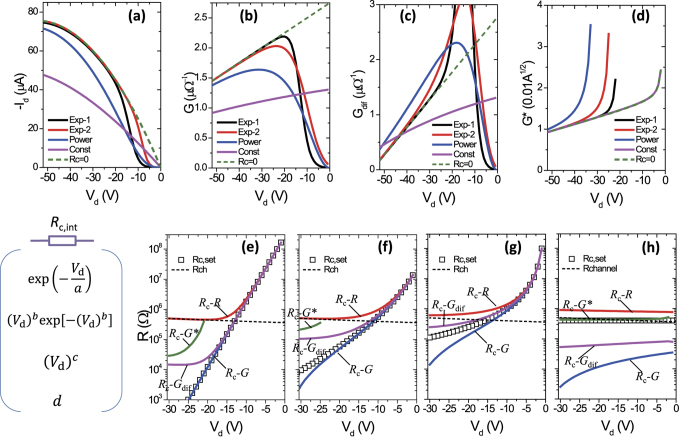
(**a**) The simulated *I*_d_–*V*_d_ characteristics for various contact properties. The extracted static output conductance *G*, dynamic output conductance *G*_dif_, and G* are depicted in (**b**–**d**), respectively. Comparison between the set values (open squares) and calculated values (lines) of *R*_c,int_ for various contact properties: (**e**) “Exp-1”, (**f**) “Exp-2”, (**g**) “Power”, and (**h**) “Constant”. The set values of *R*_ch_ (dashed lines) are also shown for reference.

The hook effect and the application to extract *R*_c,int_ are shown in Fig. 3, which depicts numerical simulations on the charge injection in OTFTs. To cover general injection behaviors, we simulated *R*_c,int_ in the forms of 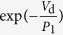 (referred to as “*Exp-1*”), 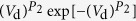 (“*Exp-2*”), 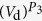 (“*Power*”), or a constant *P*_4_ (“*Const.*”) (see simulation details in experimental and SI, Part 2). The simulated *I*_d_–*V*_d_ curves are depicted in Fig. 3a and the extracted *G*, *G*_dif_, and G* are shown in Fig. 3b–d. Clearly, the hook effect appears in non-linear injections but does not appear in the linear injection (i.e., *R*_c_ = 0, dashed lines). In Fig. 3e–h, *R*_c,int_ is extracted (in lines) by Table 1 and compared with the set values *R*_c,set_ in the simulations (open squares). *G*- and *G*_dif_-functions give accurate estimations when *R*_c,int_ is strongly *V*_d_-dependent, while the G*-function does so when *R*_c,int_ is *V*_d_-independent. For comparison, values of *R*_ch_ are depicted as dashed curves and they crosses the *R*_c,set_ curves at the critical *V*_d_’. Furthermore, the *G*- and *G*_dif_-functions were examined using commercial TFT simulation tools (Silvaco Atlas) with Schottky barriers as presented in the SI (Part 3), which proves good accuracy. Importantly, the extracted resistance readily excludes the main part of the bulk resistance (*R*_c,bulk_), and therefore is mainly the interfacial contact resistance (*R*_c,int_), as discussed in the SI (part 4). In the following, we apply the method to analyze the experimental results of various OTFTs.

### Experimental results of OTFTs

**Figure 4:**
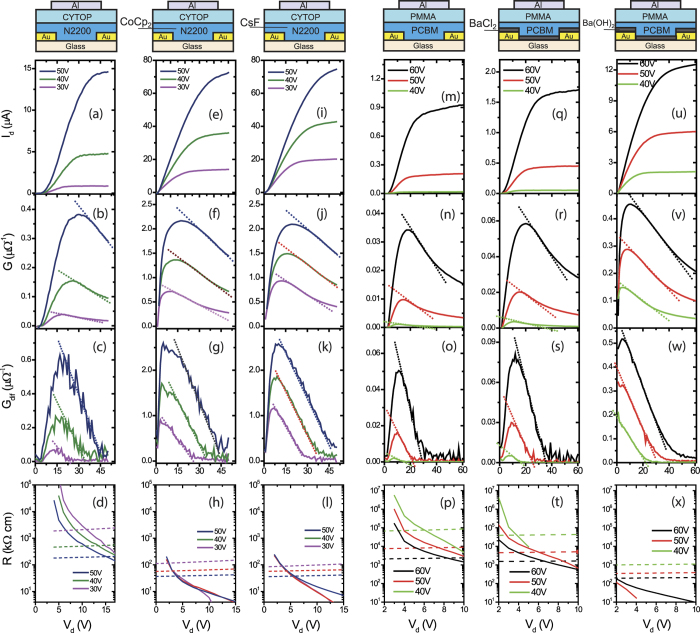
Charge injection of *n*-type FETs characterized by output characteristics, static output conductance *G*, dynamic output conductance *G*_dif_, and extracted interfacial contact resistance (*R*_c,int_) and channel resistance (*R*_ch_) for OTFTs N2200 or PCBM semiconductors. (**a**–**d**) N2200 transistors without dopant; (**e**–**h**) N2200 transistors with CoCp_2_ as bulk dopants; (**i**–**l**) N2200 transistors with CsF as bulk dopants; (**m**–**p**) PCBM transistors without charge injection layers (CILs); (**q**–**t**) PCBM transistors with BaCl_2_ as CILs; (**u**–**x**) PCBM transistors with Ba(OH)_2_ as CILs. The dotted lines in the *G* and *G*_dif_ figures illustrate the linear part. The dashed lines in the *R* figures denote the extracted channel resistance (*R*_ch_) for the corresponding voltage.

**Figure 5:**
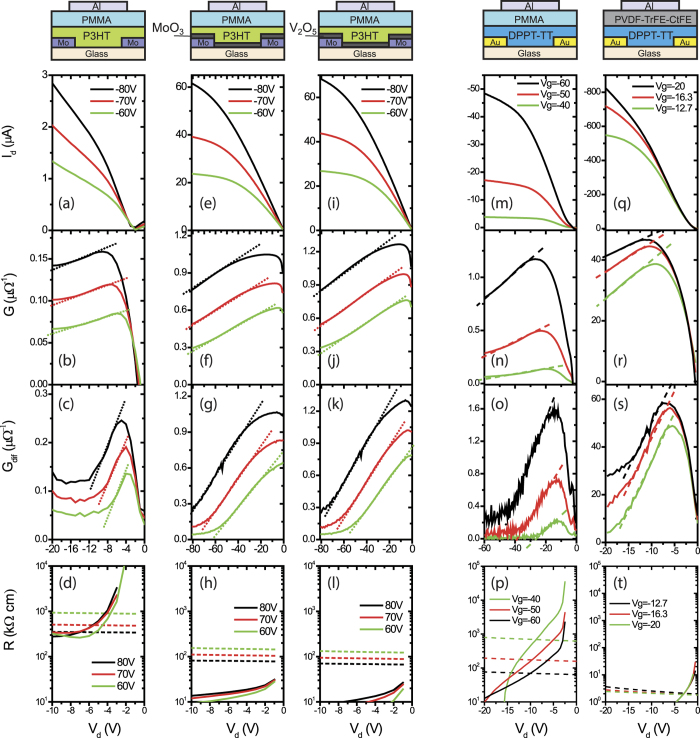
Charge injection of *p*-type FETs characterized by *G*, *G*_dif_, *R*_c,int_, and *R*_ch_ for OTFTs with P3HT or DPTT-TT semiconductors. (**a**–**d**) P3HT transistors with pure Mo electrodes; (**e**–**h**) P3HT transistors with Mo electrodes covered by MoO_3_ CILs; (**i**–**l**) P3HT transistors with Mo electrodes covered by V_2_O_5_ CILs; (**m–p**) DPPT-TT transistors PMMA dielectric; (**q**–**t**) DPPT-TT transistors PVDF-TrFE-CtFE dielectric. The dotted lines in the *G* and *G*_dif_ figures illustrate the linear part. The dashed lines in the *R* figures denote the extracted channel resistance (*R*_ch_) for the corresponding voltage.

The measurement and investigation of output characteristics of all OTFTs is shown in Fig. 4 (*n*-type) and Fig. 5 (*p*-type), and the results are summarized in Table 2. In the analysis, we intentionally skip the data next to *V*_d_ = 0 V to reduce the error induced by *V*_d_-dependent mobility, because the above methods assume that mobility is weakly dependent on *V*_d_. In all OTFTs, we observed the universal hook effect, which is manifested as follows: *G* and *G*_dif_ start from zero, raise to a maximum and then decrease linearly in all the OTFTs, regardless of the fabrication process and material of contacts, semiconductors, and dielectrics. This is the first important finding of our study.

The hook effect is alleviated by using bulk dopants, inserting charge injection layers (CILs), or even by changing dielectrics. We take the *n*-type, BC/TG OTFTs based on N2200 semiconductors for the first example (see *I*_d_, *G*, and *G*_dif_ in Fig. 4a–d). Both *G* and *G*_dif_ exhibit typical hook features followed by a linear relationship with *V*_d_ (see the dashed red lines). With organic dopant CoCp_2_ or inorganic dopant CsF in the bulk N2200, the OTFTs exhibit larger conductance (*G* and *G*_dif_ above 2 μΩ^−1^) as compared to the pristine device (below 0.6 μΩ^−1^). We use *G*-function to calculate *R*_c,int_ and *R*_ch_ and quantitatively determine *V*_d_’ (*R*_c,int_ = *R*_ch_) for each type of device in Fig. 4a–c, which signifies the transition from the injection-dominated regime to transport-dominated regime. The critical *V*_d_’ is above 15 V with pristine semiconductors, while *V*_d_’ is below 6 V with bulk dopants. As the dopants CoCp_2_ and CsF increase the carrier concentration^22^, the conductivity adjacent to the contact is enhanced, the depletion region is narrowed^12^, and the *R*_c,int_ is lowered by over 100 times.

Such improvements are also observed in OTFTs with CILs. In *n*-type OTFTs with PCBM (Fig. 4m–x), the Ba(OH)_2_ CIL reduced *R*_c,int_ as significantly as three orders of magnitude (values at 2 V). In *p*-type OTFTs with P3HT/Mo contacts (Fig. 5a–l), the solution-processed MoO_3_ and V_2_O_5_ CILs reduced *R*_c,int_ by up to two orders of magnitude, and reduced |*V*_d_’| to below −2 V. The improvement comes from reducing the energy barrier according to spectroscopic observations^23^,^24^. In addition, CILs that are strong dopants also help in *p*-type OTFTs with C8-BTBT semiconductor (SI, part 4). The FeCl_3_ CIL at the contact region increases free carriers and reduces the unoccupied traps, so that it reduced *R*_c,int_ by about 10 times and reduced *V*_d_’ to be unobservable. For reference, the extracted *R*_c,int_ for C8-BTBT OTFTs with FeCl_3_ is 6.8 kΩ·cm (*V*_d_ = −2 V) which is close to the value (8.8 kΩ·cm) extracted from TLM^25^, showing that our methods are reasonable estimations.

Besides bulk dopants and CILs, interesting results were also found in OTFTs with various dielectrics. In OTFTs with the DPPT-TT/Au contacts (Fig. 5m–t), the devices with the low-*k* PMMA dielectric (*k* = 3.5) exhibited *R*_c,int_ values of more than 1000 kΩ·cm (at *V*_d_ = −2 V) and critical *V*_d_’ in the range of −8 to −10 V, while the devices with the high-*k* PVDF-TrFE-CtFE (*k* = 10.4) featured *R*_c,int_ values two orders lower (about 20 kΩ·cm) and a much smaller critical *V*_d_’ (~−3 V). The large bulk capacitance and interfacial negative dipoles of PVDF-TrFE-CtFE induced strong electrostatic coupling to increase the carrier concentration and lowered the Fermi level towards the highest occupied molecular orbital (HOMO)^26^. The magnitude of enhancement is exceptional, considering that carrier concentration only increased about six times. The above results indicate that the hook effect generally exist as a sign of non-ohmic contact injection in OTFTs and can be used to qualitatively characterize charge injections.

**Figure 6:**
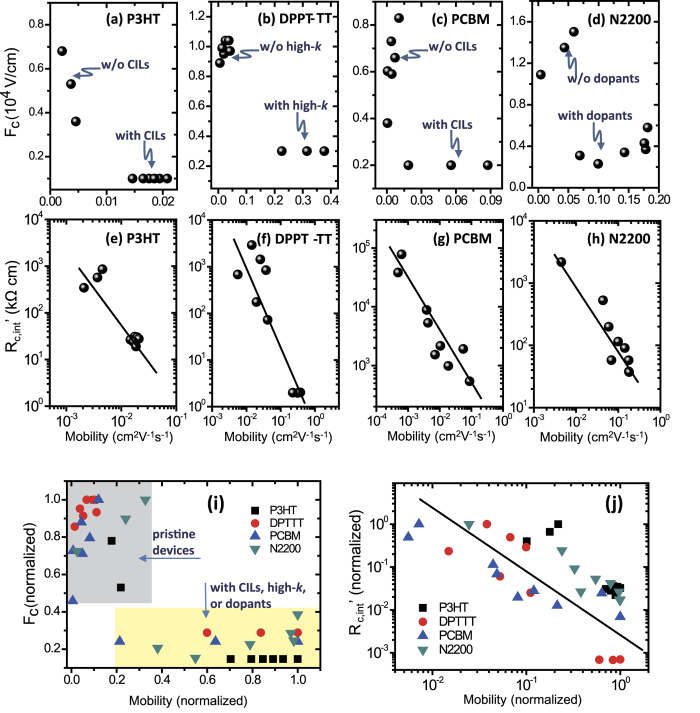
The critical field *F*_c_ (**a**–**d**) and corresponding interfacial contact resistance *R*_c,int_’ (**e**–**h**) plotted against channel mobility. The values are extracted from OTFTs (shown in Figs 4 and 5) made with P3HT (**a**,**e**), DPPT-TT (**b**,**f**), PCBM (**c**,**g**), and N2200 (**d**,**h**). In (**a**–**d**), types of devices are given by texts, and the detailed structures are shown in Figs 4 and 5. The values of *F*_c_ and *R*_c,int_’ are normalized and plotted in the (**I**,**j**) for comparison. In (**i**), grey and yellow squares are used to visualize different types of devices, comparing pristine devices and those with CIL, high-*k* dielectrics, or dopants. In (**e**–**h**,**j**), the lines are guides for the eye.

Whether the charge injection is diffusion-limited or not was firstly examined by depicting the relations between *V*_d_’, *R*_c,int_’ (*R*_c,int_ at *V*_d_’), and *μ* of OTFTs (Fig. 6a–h). The critical electric field is calculated by *F*_c_ = *V*_d_’/*L,* and for those cases where *V*_d_’ does not appear within the measurement window, we conservatively use the minimum *V*_d_ and the corresponding channel resistance as *R*_c,int_’. The calculated mobility values are lower than those reported before calculated from the saturation regime, as the values here were extracted from Table 1, whose values are close to those calculated from the linear regime. We depict normalized *F*_c_ and *R*_c,int_’ as a function of normalized *μ* for all solution-processed OTFTs (Fig. 6i,j). Here in OTFTs, it is clear that, regardless of materials, device configurations, and processing, a higher *μ* is related to a lower *F*_c_ and a lower *R*_c,int_. In detail, a three to four times higher *μ* corresponds to a three to four times lower critical field, and a 10^2^ to 10^3^ times lower *R*_c,int_. Previous studies on the relation between mobility and injection are mainly based on diodes^19^. Firstly, it indicates that tuning (or optimizing) contact injection efficiency and semiconductor mobility is always synergetic, i.e., one interacts as both cause and effect with the other. Secondly, as *R*_c,int_’ decreases as the carrier mobility increases, the results imply that charge injections in OTFTs are diffusion-limited, as Fig. 6e–h suggest a universal relation that *R*_c,int_’ decreases with *μ*.

### Diffusion-limited injection with surface recombination

**Figure 7:**
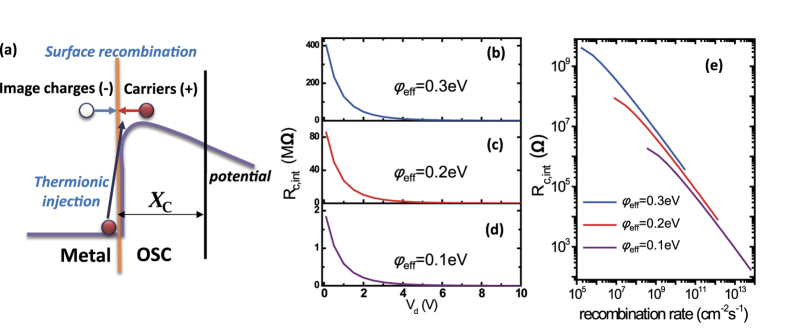
(**a**) Schematic diagram of surface recombination at the metal–organic interface (take electron current as an example). (**b**–**d**) Calculated *R*_c,int_ as a function of *V*_d_ for different injection barriers according to Eq. 8. (e) Calculated relation between *R*_c,int_ and recombination rate *r*_rec_ in various electric field for different injection barriers.

In diffusion-limited injection, we especially examine Eq. 8 because the recombination process is regarded to take place in OSCs where carriers transport mainly *via* hopping. At the metal–organic interface, the recombination can occur between the carriers and their image charges^18^, where thermionically injected carriers within *x*_C_ return to the metal surface to form the organic-to-metal current opposite to the metal-to-organic current (illustrated in Fig. 7a). This process can occur with unipolar carriers, in contrast to the recombination in the bulk of OSCs where Langevin’s recombination occurs in the form of electron–hole recombination^27^,^28^, as well as trap-assisted recombination^29^. In this scenario, the total number of charge recombination events per unit time and per unit area, i.e., the surface recombination rate *r*_rec_ (unit in cm^−2^ s^−1^), is determined by the carrier mobility together with the injection barrier. The surface recombination rate at zero field *r*_rec,0_ is (see derivation in the SI, Part 5):


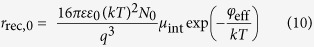


For example, when *φ*_eff_ = 0.1 eV, *μ*_int_ = 0.1 cm^2^/Vs, *N*_0_ = 10^6^, and *ε* = 3.5, the rate *r*_rec,0_ is 1.4 × 10^8^ cm^−2^ s^−1^ at 300 K, which is independent of *V*_d_ and well characterizes the interfacial conditions. When OTFT is operated at non-zero field, the rate *r*_rec_ is proportional to *r*_rec,0_ and will increase with *V*_d_, and the exact form of *r*_rec_ can be found in the SI (Part 5). Importantly, *R*_c,int_ in OTFTs will decrease with increasing *r*_rec_. The calculated *r*_rec_ and *R*_c,int_ as a function of *V*_d_ are shown in Fig. 7b–d and the correlation between *r*_rec_ and *R*_c,int_ are shown in Fig. 7e (see the SI, Part 5, for calculation details). The main features of the calculated curves are similar to the experimental results mentioned above, and hence we will use the Eq. 8 to fit the experimental data in the following.

Based on Eq. 8, for the simplicity of fitting, we assume the parameters *α* and *β* to be unity and obtain (see derivation in the SI, Part 6):


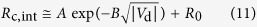


**Figure 8:**
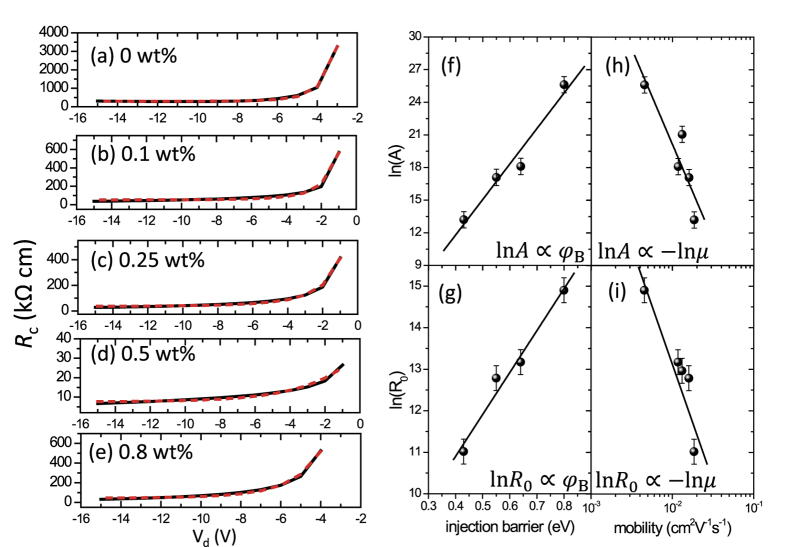
(**a**–**e**) Fitting the extracted values of R_c,int_ to Eq. 11. The extracted R_c,int_ are shown in black solid curves and the fitted curves are shown in red dashes. (**f**,**g**) Relationships between the parameter *A* and the measured injection barrier *φ*_B_ and *μ*. (**h**,**i**) Relationships between the parameter *R*_0_ and the measured injection barrier *φ*_B_ and *μ*, confirming Eqs 11, 12, 13. The measured injection barrier *φ*_B_ is calculated the difference between the work function measured from Mo/V_2_O_5_ surface and the HOMO level of P3HT.

Here, 
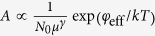
 is the most important constant related to the effective Schottky barrier *φ*_eff_, carrier mobility *μ*, and number of charge hopping sites *N*_0_; *B* is related to the local electric field and the image potential; *R*_0_ is much smaller than the first term and it should also increase exponentially with the Schottky barrier (see the SI, Part 5). The Mo/V_2_O_5_/P3HT transistors (device structure is shown in Fig. 5) were examined as in those OTFTs the work-function of contact electrode was continuously tuned by changing the thickness of the V_2_O_5_ layers. For fitting, the values of *φ*_eff_ are simply estimated by the measured injection barrier *φ*_B_, which is calculated by using the difference between the work function measured from Mo/V_2_O_5_ surface and the HOMO levels of P3HT. Reasonable fittings of Eq. 11 were obtained for the extracted *R*_c,int_ with different V_2_O_5_ layer thickness, as shown in Fig. 8a–e and Table 3. Note that when discussing injection barrier, devices spin-coated with 0 wt% to 0.5 wt% V_2_O_5_ solution were compared as the interlayers are thin and flat, except the 0.8 wt% device, which had a thick and rough insulating interlayer and the resulting extra access resistance^23^. In addition, according to Eq. 11, the fitting parameters should follow:


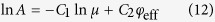



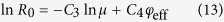


Here *C*_1_, *C*_2_, *C*_3_, and *C*_4_ are constants for *V*_d_. Importantly, Fig. 8f–i correspond well to Eqs 12 and 13 and indicate good consistency between the theoretical derivations and the experimental results, suggesting the surface recombination scenario in OTFT injections. In fact, Eq. 10 indicates that the recombination rates can be promoted by lowering the injection barrier or/and enhancing the interfacial mobility, which has been achieved in the experimental methods mentioned above, including bulk-doping, charge injection layers, and dielectric engineering.

**Figure 9:**
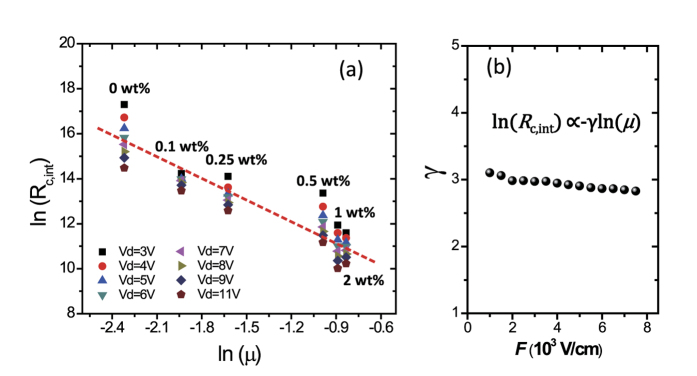
N2200 transistors with CsF dopants: (**a**) Relation between interfacial contact resistance [in terms of ln(*R*_c,int_)] and channel mobility [in terms of ln(*μ*)] for different doping concentrations (including various *V*_d_); the dashed line is a guide for the eye, indicating ln(*R*_c,int_) decreases linearly with ln(*μ*). (**b**) the parameter γ for fitting ln(*R*_c,int_) ∝−γ ln (*μ*) at different lateral fields.

To further examine the general applicability of the relation between *R*_c,int_ and carrier mobility, we analyzed the devices with n-type N2200 transistors with CsF dopants. By doping organic semiconducting layers (N2200) with dopants (CsF) in different concentrations (or weight ratios, device structure is shown in Fig. 4), the mobility of the semiconductor is tuned without changing the injection barrier and the impact of mobility on *R*_c,int_ can be solely studied. The results are shown in Fig. 9, depicting values of *R*_c,int_ of devices with different doping weight ratios of CsF (in wt%). Generally, *R*_c,int_ decreases with mobility in a power law and it is in good accordance with the theoretical prediction, which is given by the diffusion-limited injection (Eqs 6, 7, 8, 9):


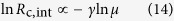


The power factor *γ* indicates how interfacial injection is affected by channel mobility, determined by the materials and device structure. Fittings of data from N2200 transistors in Fig. 9 to the above equation are generally good as the correlation coefficients for fitting are from 0.9006 to 0.9841. Moreover, the values of power factor *γ* are around 3.0 at different drain field determined by *V*_d_ values, indicating that the assumption that mobility is almost independent of lateral electric field in the studied region. The results again confirm the above theory on the diffusion-limited injection where *R*_c,int_ follows mobility.

## Discussions

Charge injection of OTFTs with various semiconductors and contact materials was investigated systematically. According to our calculations, real ohmic contact can hardly be achieved in common OTFTs with their low carrier mobilities and low carrier concentrations. Consistently, the hook effect signifying non-ohmic contact was experimentally found to be universal in OTFTs. For different non-ohmic injection mechanisms, different forms of interfacial contact resistance have been theoretically derived, which can be summarized into a general applicable form. We also developed a series of functions to analyze the interfacial contact resistances and used them to study OTFTs with a broad range of materials.

The experimental results of TFTs confirm the proposed form of interfacial contact resistance, and strongly support that OTFTs universally suffer from diffusion-limited injection, where the interfacial contact resistance generally decreases with carrier mobility. The injection process is also probably accompanied by a surface recombination process at the metal-organic interfaces. The surface recombination rate determines the injection current and has been promoted by bulk-doping, contact engineering with charge injection layers and dopant layers, and even dielectric engineering with high-*k* dielectric materials. As a result, the critical voltage |*V*_d_’|, signifying the transition from the injection-limited to the field-effect transport regime, has also been reduced from generally over 10 V towards zero.

## Methods

### Fabrication and characterizations of OTFTs

All the fabrication and characterization of OTFTs were carried out in an N_2_ atmosphere unless stated otherwise.

#### N2200 OTFTs

A top-gate/bottom-contact (TG/BC) structure was fabricated on a glass substrate. The bottom contact electrodes were Cr/Au (3/27 nm). The semiconductor is N2200 or N2200 with CoCp2 and CsF doping. The N2200 film deposited by spin casting from the *p*-xylene solution and dried at 110 °C (30 nm thick). The bulk dopants were mixed with N2200 solution in the weight ratio in 0.025%, 0.5%, 1.0%, and 2.0% before spin-coating. The gate dielectric is CYTOP (500 nm, deposited by spin casting and annealed at 130 °C for 1 hour). The top gate electrode is Al (50 nm, deposited by thermal evaporation in high vacuum). The channel length is 10 μm and the channel width is 1 mm.

#### PC_61_BM OTFTs

The TFTs in TG/BC structure was fabricated on a glass substrate. The contact electrodes are Ni/Au (3/12 nm) deposited by thermal evaporation and patterned by photolithography. For the interlayers, Ba(OH_2_) and Ba(Cl_2_) (from Sigma-Aldrich) were dissolved in methanol (2 mg/ml) and spin-coated onto the Au patterned electrodes (4000 rpm)^24^. The *n*-channel semiconductor PC_61_BM (from Nano-C) was dissolved in anhydrous chlorobenzene (10 mg/ml), spin-coated at 2000 rpm, and annealed at 110 °C for 20 min. The top-gate dielectric layer was deposited by spin-coating PMMA (MW = 120 kD, from Sigma-Aldrich) dissolved in n-butyl acetate (80 mg/ml) followed by annealing at 80 °C for 30 min. The top-gate electrodes were fabricated by the thermal deposition of aluminum (Al) *via* metallic shadow masks (50 nm thick). The channel length is 20 μm and the channel width is 1 mm.

#### P3HT OTFTs

A TG/BC structure was fabricated on a glass substrate. The bottom contact electrodes were fabricated by sputtering Ni/Mo (3/13 nm) and conventional photolithography process to define patterns for source and drain electrodes. For the MoO_3_ interlayers, the ammonium molybdate (NH_4_)_2_MoO_4_ in H_2_O solution (0.8 wt% of MoO_3_) was spin-coated and annealed at 150 °C for 10 min in air^30^. For V_2_O_5_ interlayers, the ammonium vanadate ((NH_4_)_3_VO_4_) solution of different concentration (0.1–0.5 wt% of V_2_O_5_) was spin-coated and then annealed at 150 °C for 20 min in air^23^. Then semiconducting polymer rr-P3HT (from Sigma-Aldrich) dissolved in anhydrous dichlorobenzene (DCB, 10 mg/ml) was spin-coated and annealed at 150 °C for 30 min. The top-gate dielectric layer PMMA and the top Al electrodes were deposited as described above. The channel length is 10 μm, and channel width *W* is 1 mm.

#### C8-BTBT OTFTs

The bottom-gate electrodes are Ti/Au (3/37 nm, formed by vacuum evaporation), the gate dielectric was parylene (290 nm, deposited by chemical vapor deposition), the semiconductor was C8-BTBT (40 nm film, evaporated at 0.01 nm/s, Nippon Kayaku), and the top contact electrodes were pristine Au (50 nm) or FeCl3/Au (0.3/40 nm, evaporated at 0.01 nm/s). The channel length is 350 μm and the channel width is 1000 μm.

#### DPPT-TT OTFTs

A TG/BC structure was fabricated on a glass substrate with the bottom contact electrodes Ni/Au (3/12 nm) deposited as stated above. The *p*-channel semiconductor DPPT-TT was spin-coated from anhydrous chlorobenzene solution (10 mg/ml) and annealed at 200 °C for 30 min. The gate dielectric layer is PMMA, PS, or PVDF-TrFE-CtFE (formed on top of the DPPT-TT layer by spin-coating). The capacitance per unit area is 6.2 nF/cm^2^ for PMMA, 4.6 nF/cm^2^ for PS, and 63.72 nF/cm^2^ for PVDF-TrFE-CtFE, respectively. The top gate electrode is Al (50 nm, by thermal deposition). The channel length is 10 μm and channel width *W* is 1 mm. In the figure for DPPT-TT (b,f), the data of OTFTs using polystyrene (PS) dielectrics are also included.

#### Characterizations

The electrical characteristics of OTFTs were measured with an Agilent 4156A Semiconductor Parameter Analyzer in an N_2_ atmosphere. *L* and *W* are channel length and width, respectively, *μ* is carrier mobility in channel, *V*_g_ is gate-voltage, and *V*_th_ is the threshold voltage.

### Calculation and simulation of interfacial contact resistance

Data in Fig. 2 in the main text were calculated by simulating the *I*_d_-*V*_d_ characteristics in the linear regime using the equation (Fig. 2):


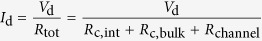


Here, 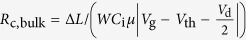 and 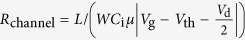. The values of Δ*L* do not affect the extracted *R*_c,int_. Then the values of *R*_c,int_ are set in the following forms: 
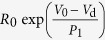
 (referred to as “*Exp-1*”), 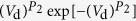 (“*Exp-2*”), 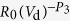 (“*Power*”), *P*_4_ (“*Const*”). In the calculation and simulation, *V*_d_ ranges from 0 V to −30 V with a step of −1 V. In Fig. 2, the set values of *R*_c,int_ and calculated *R*_c,int_ extracted by Table 1 are compared. The values of calculation and simulation parameters can be found in SI, Part 2.

### Calculation and simulation of surface recombination rate

The surface recombination rate *r*_rec_ can be calculated as the product of the surface charge density *n* and the surface recombination velocity *S*. The voltage across the interface *V*_a_ and local electric field *ϵ* at the interface and are affected by *V*_d_. Assume the former is 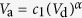 and the latter is 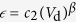, and charge mobility near the interface follows the field-effect mobility 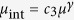. Then *r*_rec_ can be expressed by (unit in cm^−2^s^−1^) (Fig. 7)


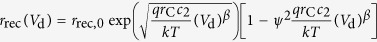


Here *ψ* is a weak function of *ϵ* (see SI for details). The detailed derivation and values of parameters in calculations and simulations can be found in SI, Part 5.

## Additional Information

**How to cite this article**: Liu, C. *et al.* Universal diffusion-limited injection and the hook effect in organic thin-film transistors. *Sci. Rep.* **6**, 29811; doi: 10.1038/srep29811 (2016).

## Supplementary Material

Supplementary Information

## Figures and Tables

**Table 1:** The derived functions to estimate *R*_c,int_ from output characteristics.

	*R*-function	*G*-function	*G*_dif_ -function	G^*^-function
Key parameter	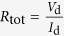	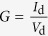	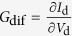	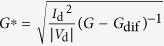
μ	**—**	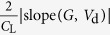	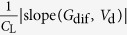	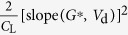
*V*_th_	**—**	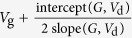	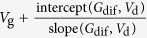	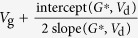
*R*_ch_	**—**	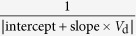	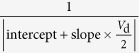	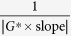
*R*_c_	~*R*_tot_	(*R*_tot_ − *R*_ch_)	(*R*_tot_ − *R*_ch_)	(*R*_tot_ − *R*_ch_)

The expressions are applicable for both *p*- and *n*-type devices (note that *V*_d_ and *I*_d_ have the same sign). The extrapolated parameters are expressed in “slope (y, x)” and “intercept (y, x)”. In calculations, we defined *G*_dif_ as *G*_dif_(*n*) = [*I*_d_(*n*) − *I*_d_(*n* − 1)]/[*V*_d_(*n*) − *V*_d_(*n* − 1)], where *n* is the number of measured data. Here it is assumed that *R*_c,bulk_ can be ignored and, when it is not ignorable, it is included in the extracted *R*_ch_.

**Table 2:** Characteristic parameters of the OTFTs investigated in this study.

Contact metal	Semiconductor	Dielectric	C_i_	V_g_	μ	V_d′_
Mo	P3HT	PMMA	6.2 × 10^−9^	−60.0	2.1 × 10^−3^	−3.6
				−70.0	3.7 × 10^−3^	−5.4
				−80.0	4.5 × 10^−3^	−7.0
Mo/MoO3				−60.0	1.8 × 10^−3^	−2.0
				−70.0	1.9 × 10^−2^	−2.0
				−80.0	2.1 × 10^−2^	−2.0
Mo/V2O5				−60.0	1.5 × 10^−2^	−2.0
				−70.0	1.7 × 10^−2^	−2.0
				^−^80.0	1.9 × 10^−2^	−2.0
Au	DPTTT	PMMA	6.2 × 10^−9^	−40.0	5.6 × 10^−3^	−8.5
				−50.0	2.0 × 10^−2^	−9.5
				−60.0	4.2 × 10^−2^	−10.0
		PS	4.6 × 10^−9^	−60.0	1.4 × 10^−2^	−10.0
				−70.0	2.5 × 10^−2^	−10.0
				−80.0	3.7 × 10^−2^	−10.5
		PVDF-TrFE-CTFE	6.4 × 10^−9^	−12.7	3.8 × 10^−1^	−3.0
				−16.3	3.2 × 10^−1^	−3.0
				−20.0	2.3 × 10^−1^	−3.0
Au	PCBM	PMMA	5.3 × 10^−9^	40.0	6.3 × 10^−4^	6.0
				50.0	3.9 × 10^−3^	7.5
				60.0	1.1 × 10^−2^	8.3
Au/BaCl_2_				40.0	4.9 × 10^−4^	3.8
				50.0	4.3 × 10^−3^	6.0
				60.0	7.1 × 10^−3^	6.5
Au/Ba(OH)_2_				40.0	1.9 × 10^−2^	2.0
				50.0	5.6 × 10^−2^	2.0
				60.0	8.8 × 10^−2^	2.0
Au	N2200	CYTOP	3.7 × 10^−9^	30.0	4.5 × 10^−3^	11.0
				40.0	4.3 × 10^−2^	13.5
				50.0	5.9 × 10^−2^	15
	N2200:CoCp2			30.0	9.9 × 10^−2^	2.2
				40.0	6.9 × 10^−2^	3.0
				50.0	1.8 × 10^−1^	3.7
	N2200:CsF			30.0	1.4 × 10^−1^	3.5
				40.0	1.8 × 10^−1^	4.2
				50.0	1.8 × 10^−1^	5.6

The uncertainty of calculated *μ* and *V*_d_ is estimated to be less than 30%.

**Table 3:** Fitting results of *R*_c,int_ to Equation 11.

	0 wt%	0.1 wt%	0.25 wt%	0.5 wt%	0.8 wt%
***A*** (*kΩ* cm)	1.33 × 10^7^	7.26 × 10^3^	2.65 × 10^3^	5.39 × 10^1^	1.40 × 10^5^
***R***_***0***_ (*kΩ* cm)	2.96 × 10^2^	5.26 × 10^1^	3.57 × 10^1^	6.09 × 10^0^	4.27 × 10^1^
***B***	0.21	0.38	0.51	1.00	0.35
*Correlation coefficient*	0.9986	0.9895	0.9941	0.9927	0.9928

The device structure and fitting curves are shown in Fig. 8. The correlation coefficient *r* for fitting are also given.
