# Supplementary material for: Universal diffusion-limited injection and the hook effect in organic thin-film transistors
Source: Sci Rep. 2016 Jul 21;6:29811. doi: 10.1038/srep29811 (PMC4954995; doi:10.1038/srep29811)
Supplement: Supplementary Information [file srep29811-s1.pdf]

Supplementary information

Universal diffusion-limited injection and the hook effect in organic thin-film transistors

Chuan Liu[1], Gunel Huseynova[2], Yong Xu[2], Dang Xuan Long[2], Won-Tae Park[2], Xuying Liu[3], Takeo Minari[3], Yong-Young Noh[2]

Part 1. Derivation of Table 1 in the main texts

Part 2. Simulation methods and parameters for Figure 2

Part 3. Device simulation by Silvaco Atlas; comparison between G-, G_{dif}-, and TLM methods

Part 4. The meaning of extracted $R_{\text{c,int}}$; Data of C8-BTBT devices

Part 5. Derivation of general surface recombination rate

Part 6. Derivation of $R_{\text{c,int}}$ and Equation 16 in the main text

Part 1. Derivation of Table 1 (functions for extracting contact resistance from output characteristics)

Basic assumption: mobility is weakly dependent on V_d in the studied region (the mobility extracted here is an average of all V_d region). In the following a parameter is used, $C_L = \frac{C_{ox}W}{L}$.

I. *R*-function (for simple examination of R_C)

$$R_{tot} = \frac{V_d}{I_d} = R_c + \frac{L}{W}R_{sh} \quad \text{(s1-1)}$$

When V_d is small and $R_c \gg \frac{L}{W}R_{sh}$,

$$R_c \sim R_{tot} \quad \text{(s1-2)}$$

However, measured data can be noisy and this fitting does not often show clear information.

II. *G*-function (for general cases to extract R_C)

II-1. The total output conductance is defined by,

$$G = \frac{I_d}{V_d} = \frac{1}{R_{tot}} = \frac{1}{R_c + \frac{L}{W}R_{sh}} \quad \text{(s1-3)}$$

$$R_{sh} = \frac{1}{C_{ox}\mu\left|V_g - V_{th} - \frac{V_d}{2}\right|} \quad \text{(s1-4)}$$

II-2. As V_d increases, R_{sh} increases and R_c generally decreases due to the fundamental properties of diode and image lowering. As V_d increases, $R_c \ll \frac{L}{W}R_{sh}$

$$G = \frac{1}{R_c + \frac{L}{W}R_{sh}} \approx \frac{1}{\frac{L}{W}R_{sh}} = C_L\mu\left|V_g - V_{th} - \frac{V_d}{2}\right| \quad \text{(s1-5)}$$

II-3. Therefore, when V_d is large, G decreases linearly with V_d,

$$\text{slope}(G, \text{Vd}) = \frac{\partial G}{\partial V_d} = \frac{-C_{ox}W\mu}{2L} = -\frac{C_L\mu}{2} \quad \text{(s1-6)}$$

$$\text{intercept}(G, \text{Vd}) = \frac{WC_{ox}\mu}{L}\left(V_g - V_{th}\right) = C_L\mu\left(V_g - V_{th}\right) \quad \text{(s1-7)}$$

$$\mu = \frac{2L}{C_{ox}W}|\mathrm{slope}(G, V_d)| = \frac{2}{C_L}|\mathrm{slope}(G, V_d)| \quad \text{(s1-8)}$$

$$(V_g - V_{th}) = -\frac{\mathrm{intercept}(G, V_d)}{2\ \mathrm{slope}} \quad \text{(s1-9)}$$

$$R_{sh}\frac{L}{W} = \frac{1}{\mathrm{intercept} + \mathrm{slope} \times V_d} \quad \text{(s1-10)}$$

Using this equation can directly derive the mobility at certain V_g from one output curve, and it is free from contact resistance.

II-4. Then, use this mobility to extract contact resistance:

$$R_c = \frac{V_d}{I_d} - R_{sh}\frac{L}{W} = \frac{V_d}{I_d} - \frac{L}{W}\frac{1}{C_{ox}\mu\left|V_g - V_{th} - \frac{V_d}{2}\right|} = \frac{1}{G} - \frac{1}{\mathrm{intercept} + \mathrm{slope} \times V_d} \quad \text{(s1-11)}$$

III. G_{dif}-function (for the cases that G is not sensitive to V_d)

III-1. The differential output conductance is defined by,

$$G_{dif} = \frac{\partial I_d}{\partial V_d} = \frac{\partial\left(\frac{V_d}{R_{tot}}\right)}{\partial V_d} \quad \text{(s1-12)}$$

$$\sigma_{sh} = \frac{1}{R_{sh}} = C_{ox}\mu\left|V_g - V_{th} - \frac{V_d}{2}\right| \quad \text{(s1-13)}$$

$$G_{dif} = \frac{\partial\left[V_d/\left(R_c + R_{sh}\frac{L}{W}\right)\right]}{\partial V_d} = \frac{1}{\left(R_c + R_{sh}\frac{L}{W}\right)} - \frac{V_d}{\left(R_c + R_{sh}\frac{L}{W}\right)^2}\frac{\partial\left(R_c + R_{sh}\frac{L}{W}\right)}{\partial V_d} \quad \text{(s1-14)}$$

III-2. As V_d increases, $R_c \ll \frac{L}{W}R_{sh}$, G_{dif} decreases linearly with V_d

$$G_{dif} \approx \frac{1}{R_{sh}\frac{L}{W}} - \frac{V_d}{\left(R_{sh}\frac{L}{W}\right)^2}\frac{\partial\left(R_{sh}\frac{L}{W}\right)}{\partial V_d} = \left(\frac{WC_{ox}\mu}{L}\right)(V_g - V_{th}) - \left(\frac{WC_{ox}\mu}{L}\right)V_d \quad \text{(s1-15)}$$

III-3. Therefore, when V_d is large, G_{dif}-V_d is linear,

$$\mathrm{slope}(G_{dif}, V_d) = \frac{\partial G_{dif}}{\partial V_d} = \frac{-WC_{ox}\mu}{L} = -C_L\mu \quad \text{(s1-16)}$$

$$\mathrm{intercept}(G_{dif}, V_d) = \frac{WC_{ox}\mu}{L}(V_g - V_{th}) = C_L\mu(V_g - V_{th}) \quad \text{(s1-17)}$$

$$\mu = \frac{L}{C_{ox}W}|\mathrm{slope}(G_{dif}, V_d)| = \frac{1}{C_L}|\mathrm{slope}(G_{dif}, V_d)| \quad \text{(s1-18)}$$

$$\left(V_g - V_{th}\right) = -\frac{\text{intercept}(G_{dif}, V_d)}{\text{slope}} \quad \text{(s1-19)}$$

$$R_{sh}\frac{L}{W} = \frac{1}{\text{intercept}+\text{slope}\times\frac{V_d}{2}} \quad \text{(s1-20)}$$

III-4. So the contact resistance can be extracted as,

$$R_c = \frac{V_d}{I_d} - R_{sh}\frac{L}{W} = \frac{V_d}{I_d} - \frac{L}{W}\frac{1}{C_{ox}\mu\left|V_g - V_{th} - \frac{V_d}{2}\right|} = \frac{1}{G} - \frac{1}{\text{intercept}+\text{slope}\times\frac{V_d}{2}} \quad \text{(s1-21)}$$

IV. G^*-function (for the cases that R_c is almost constant with V_d)

IV-1. The differential output conductance is expressed by **s12-s14**.

IV-2. As V_d increases, $\frac{\partial R_c}{\partial V_d} \ll \frac{\partial \frac{L}{W} R_{sh}}{\partial V_d}$, $R_{sh} = \frac{1}{C_{ox}\mu|V_g - V_{th} - V_d/2|}$. So we have

$$G_{dif} \cong \frac{I_d}{V_d} - \frac{I_d{}^2}{V_d}\frac{\partial\left(R_{sh}\frac{L}{W}\right)}{\partial V_d} = \frac{I_d}{V_d} - \frac{I_d{}^2}{V_d}\left(\frac{L}{W}\right)\left(\frac{1}{C_{ox}\mu\left|V_g - V_{th} - \frac{V_d}{2}\right|}\right)^2\left(\frac{C_{ox}\mu}{2}\right)$$

$$\left(\left|V_g - V_{th} - \frac{V_d}{2}\right|\right)^2\left(\frac{2C_{ox}W}{L}\mu\right) = \frac{I_d{}^2}{V_d}\left(\frac{I_d}{V_d} - G_{dif}\right)^{-1} \quad \text{(s1-22)}$$

$$G^* = \sqrt{\frac{I_d{}^2}{V_d}\left(\frac{I_d}{V_d} - G_{dif}\right)^{-1}} = \sqrt{\frac{2C_{ox}W}{L}\mu}\left|V_g - V_{th} - \frac{V_d}{2}\right| = \sqrt{2C_L\mu}\left|V_g - V_{th} - \frac{V_d}{2}\right| \quad \text{(s1-23)}$$

IV-3. Therefore, when V_d is large, G^*-V_d is linear,

$$\text{slope}(G^*, V_d) = -\frac{1}{2}\sqrt{\frac{2C_{ox}W}{L}\mu} = -\sqrt{\frac{C_L\mu}{2}} \quad \text{(s1-24)}$$

$$\text{intercept}(G^*, V_d) = \sqrt{\frac{2C_{ox}W}{L}\mu}\left(V_g - V_{th}\right) = \sqrt{2C_L\mu}\left(V_g - V_{th}\right) \quad \text{(s1-25)}$$

$$\mu = \frac{2}{C_L}[\text{slope}(G^*, V_d)]^2 \quad \text{(s1-26)}$$

$$\left(V_g - V_{th}\right) = -\frac{\text{intercept}(G^*, V_d)}{2\ \text{slope}} \quad \text{(s1-27)}$$

$$R_{sh}\frac{L}{W} = \frac{1}{C_L\mu\left|V_g - V_{th} - \frac{V_d}{2}\right|} = -\frac{1}{G^*}\frac{1}{\text{slope}(G^*, V_d)} \quad \text{(s1-28)}$$

IV-4. So R_c can be extracted as

$$R_{\mathrm{c}} = \frac{V_{\mathrm{d}}}{I_{\mathrm{d}}} - R_{\mathrm{sh}}\frac{L}{W} = \frac{V_{\mathrm{d}}}{I_{\mathrm{d}}} - \frac{L}{W}\frac{1}{C_{\mathrm{ox}}\mu\left|V_{\mathrm{g}}-V_{\mathrm{th}}-\frac{V_{\mathrm{d}}}{2}\right|} = \frac{V_{\mathrm{d}}}{I_{\mathrm{d}}} + \frac{1}{G^{*}}\frac{1}{\mathrm{slope}(G^{*},V_{\mathrm{d}})} \quad \text{(s1-29)}$$

Part 2. Simulation methods and parameters (Figure 2 in the main texts)

The I_d-V_d characteristics in the linear regime were simulated by the following set of equations:

$$I_{\mathrm{d}} = \frac{V_{\mathrm{d}}}{R_{\mathrm{tot}}} = \frac{V_{\mathrm{d}}}{R_{\mathrm{c}}+R_{\mathrm{channel}}} = \frac{V_{\mathrm{d}}}{R_{\mathrm{c,int}}+R_{\mathrm{c,bulk}}+R_{\mathrm{channel}}} \quad \text{(s2-1)}$$

$$R_{\mathrm{c,bulk}} = \frac{\Delta L}{W}\frac{1}{C_{\mathrm{i}}\mu\left|V_{\mathrm{g}}-V_{\mathrm{th}}-\frac{V_{\mathrm{d}}}{2}\right|} \quad \text{(s2-2)}$$

$$R_{\mathrm{channel}} = \frac{L}{W}\frac{1}{C_{\mathrm{i}}\mu\left|V_{\mathrm{g}}-V_{\mathrm{th}}-\frac{V_{\mathrm{d}}}{2}\right|} \quad \text{(s2-3)}$$

The expressions of $R_{c,int}$ have the following models,

$$\left(R_{\mathrm{c,int}}\right)_1 = R_0\exp\left(\frac{V_0-V_{\mathrm{d}}}{P_1}\right) \quad \text{(s2-4)}$$

$$\left(R_{\mathrm{c,int}}\right)_2 = (V_{\mathrm{d}})^{P_2}\exp[-(V_{\mathrm{d}})^{P_2}] \quad \text{(s2-5)}$$

$$\left(R_{\mathrm{c,int}}\right)_3 = R_0(V_{\mathrm{d}})^{-P_3} \quad \text{(s2-6)}$$

$$\left(R_{\mathrm{c,int}}\right)_4 = P_4 \quad \text{(s2-7)}$$

These forms are to consider the typical cases given by Eq. 9 in the main text. The parameters for simulations are list in **Table S1** (*p*-type device).

Table S1. Parameters for simulation shown in **Figure 2**.

L **(μm)**	***W*** **(μm)**	**C_i (F/cm²)**	**μ (cm²/Vs)**	**V_g (V)**	**V_{th} (V)**
20	1000	1e-8	0.1	-60	-5

R_0 (Ω)	V_0 (V)	P_1 (V)	P_2	P_3	P_4 (Ω)
1e8	2	2	0.5	2	4e4

In the simulation, V_d ranges from 0 V to -60 V with a step of -1 V (**Figure 2** in the main text shows the data from 0 to -30V). The values of ΔL does not affect the calculated $R_{c,int}$, and we compare the set values of $R_{c,int}$ and calculated $R_{c,int}$ in **Figure 2**.

Part 3 Using Silvaco ATLAS to compare *G*- and G_{dif}- functions with TLM

Simulation methods are similar to that introduced in Ref. 10 in the main text.

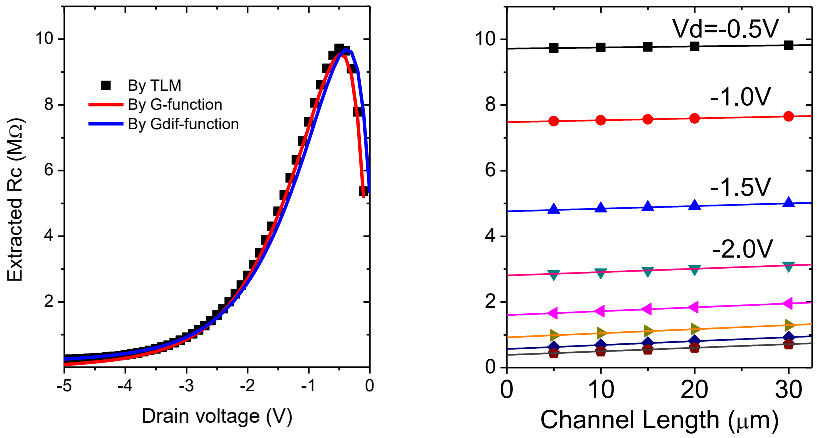

Figure S1 Comparison of R_c extracted by TLM, *G*-function and G_{dif}-functions.

Part 4 R_c extracted by Table 1 is the interfacial resistance

Usually R_c is a combination of the interfacial injection resistance ($R_{c,int}$) and the bulk injection resistance ($R_{c,bulk}$) modulated by the gate field,[1] in which case we can still use the *G*-function method. We express $R_{c,bulk}$ in the sum of an extended channel term ($R_{sh}\Delta L/W$) and a remainder term $\Delta R(V_d, V_g)$, as found in many inorganic and organic TFTs[2] [3]. This is shown as:

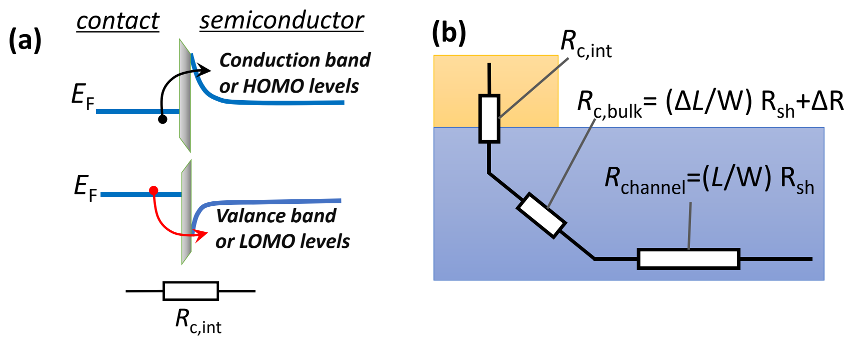

Figure S2. Illustration of interfacial contact resistance and the total resistance.

When the bulk resistance contributes significantly to R_c, i.e. ΔL. Strictly speaking,

$$R_c = R_{c,int}(V_d, V_g) + R_{c,bulk}(V_d, V_g) = R_{c,int}(V_d, V_g) + \left[\frac{\Delta L}{C_{ox}\mu W\left|V_g - V_{th} - \frac{V_d}{2}\right|} + \Delta(V_d, V_g)\right] \quad \text{(s4-1)}$$

If the remainder $\Delta(V_d, V_g)$ is far from zero, then the above calculated R_c is in fact $R_{c,int}+\Delta(V_d, V_g)$. Also, calculation of V_{th} is still valid. Usually we can assume the remainder term $\Delta(V_d, V_g)\sim 0$, because TLM converges at different V_g in many OTFTs.[2]

$$R_{tot} = R_{c,int}(V_d, V_g) + R_{c,bulk}(V_d, V_g) + \frac{L}{W}R_{sh} = R_{c,int}(V_d, V_g) + \frac{L+\Delta L}{W}R_{sh} \quad \text{(s4-2)}$$

So in all the above calculations, L should be replaced with $\Delta L+L$. The calculated mobility (μ) becomes an underestimated value, and the true mobility is:

$$\mu_0 = \frac{(L+\Delta L)}{L}\mu \quad \text{(s4-3)}$$

By changing from R_c to $R_{c,int}$, the above calculation is still valid for all the three methods. Note that the extracted value now becomes [$R_{c,int}+\Delta R(V_d, V_g)$]. If the extracted values are strongly V_g-dependent rather than V_d, the injection should be mainly determined by V_g, indicating that TFT is dominated by bulk injection. Otherwise, if they are mainly affected by V_d, then $\Delta R(V_d, V_g)$ can be ignored because $\Delta R(V_d, V_g)$ is a part of $R_{c,bulk}$ modulated by V_g. In such case, we can consider the extracted R_c by Eq. 7 as a good approximation of the *interfacial resistance* $R_{c,int}$. This is an important feature of the proposed method different from traditional transfer-length method (TLM).

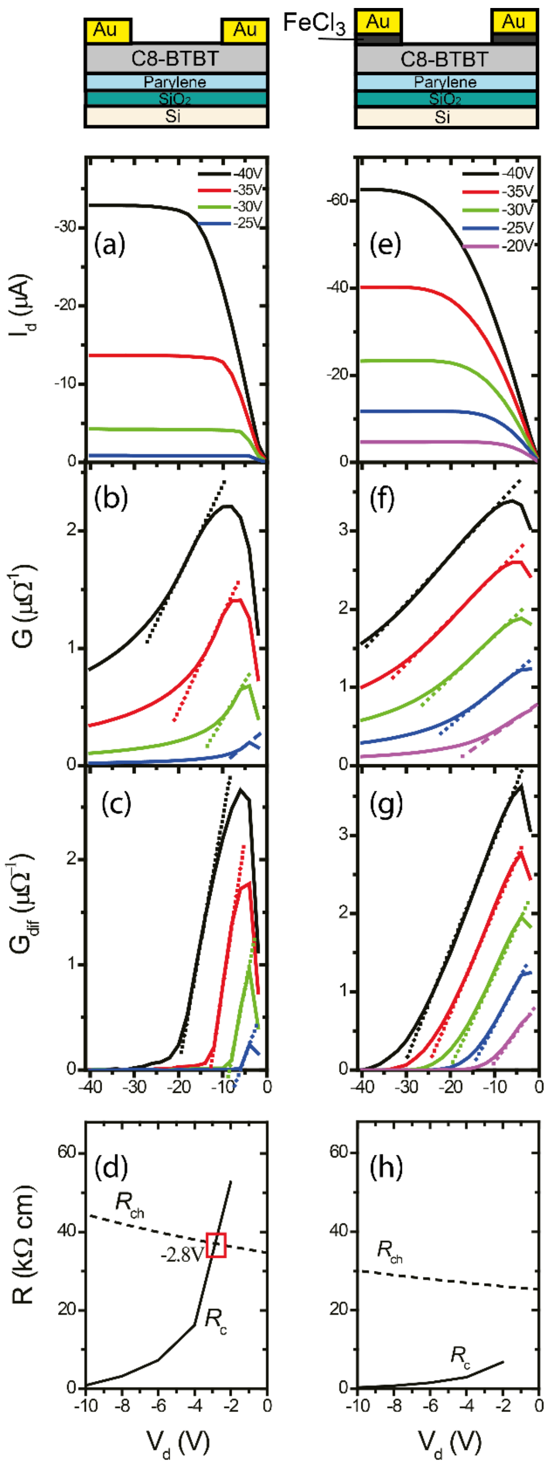

Figure S3 Charge injection of *p*-type FETs characterized by G, G_{dif}, $R_{c,int}$, and R_{ch} for OTFTs with C8-BTBT semiconductor. (a-d) C8-BTBT transistors with pure Au electrodes; (e-h) C8-BTBT transistors with Au electrodes covered by $FeCl_3$ CILs.

Part 5. Derivation of general surface recombination rate

The surface recombination rate r_{rec} can be calculated as follows.

(1) Using Langevin's recombination model, carriers recombine with the mirror charges when thermal energy kT reaches the carrier–image binding energy at a distance of $x_{\mathrm{C}}=r_{\mathrm{C}}/4$, where r_{C} is the Coulomb radius $r_{\mathrm{C}}= q^2/4\pi\varepsilon\varepsilon_0 kT$. So by using $l= x_{\mathrm{C}}$ and $D=(kT/q)\mu_{\mathrm{int}}$ (μ_{int} is the carrier mobility near the interface), we can calculate the surface recombination velocity (unit in cm s^{-1}) as,

$$S_0 = \frac{l}{\tau} = \frac{D}{l} = \frac{kT}{qx_{\mathrm{C}}}\mu_{\mathrm{int}} = \frac{16\pi\varepsilon\varepsilon_0(kT)^2}{q^3}\mu_{\mathrm{int}} \quad \text{(s5-1)}$$

This form is exactly the same with the one derived by Scott et al[4]. In addition, the mean free time τ during which the carriers travel a distance of l before recombination is

$$\tau = \frac{l^2}{D} = \left(\frac{q^2}{16\pi\varepsilon\varepsilon_0 kT}\right)^2 \frac{q}{kT}\frac{1}{\mu_{\mathrm{int}}} = \frac{1}{P} \quad \text{(s5-2)}$$

Here P is the probable recombination events that one carrier can encounter in a unit time (i.e. in one second).

(2) Then the surface recombination rate $r_{\mathrm{rec}}(V_{\mathrm{d}})$ at the contact interface of OTFT, defined as the number of recombination between carriers and the image-charges per unit time (in one second) and per unit area (in one cm^2), is the product of the surface charge density n and the surface recombination velocity S . The voltage across the interface V_{a} and local electric field ϵ at the interface and are affected by V_{d}. Assume the former is $V_{\mathrm{a}} = c_1(V_{\mathrm{d}})^{\alpha}$ and the latter is $\epsilon = c_2(V_{\mathrm{d}})^{\beta}$, and charge mobility near the interface follows the field-effect mobility $\mu_{\mathrm{int}} = c_3\mu^{\gamma}$. It is expressed by (unit in $cm^{-2}s^{-1}$),[4]

$$r_{\mathrm{rec}}(V_{\mathrm{d}}) = nS = \left[4\psi^2 N_0 \exp\left(-\frac{\varphi_{\mathrm{eff}}}{kT}\right)\exp\left(\sqrt{\frac{qr_{\mathrm{C}}c_2}{kT}(V_{\mathrm{d}})^{\beta}}\right)\right] \times \left[\frac{1}{4}\left(\frac{16\pi\varepsilon\varepsilon_0(kT)^2}{q^3}\mu_{\mathrm{int}}\right)\left(\frac{1}{\psi^2} - \frac{qr_{\mathrm{C}}c_2}{kT}(V_{\mathrm{d}})^{\beta}\right)\right] \quad \text{(s5-3)}$$

$$\psi(f) = f^{-1} + f^{-1/2} - f^{-1}\sqrt{(1+2f^{1/2})} \quad \text{(s5-4)}$$

$$f = \frac{qr_{\mathrm{C}}}{kT}\epsilon \quad \text{(s5-5)}$$

Here ψ is a weak function of ϵ [4]. So at zero field (V_d=0 and $\psi = 1$), $r_{\rm rec,0}$ is,

$$r_{\rm rec,0} = n_0 S_0 = \left[N_0 \exp\left(-\frac{\varphi_{\rm eff}}{kT}\right)\right] \times \left[\frac{16\pi\varepsilon\varepsilon_0 (kT)^2}{q^3}\mu_{\rm int}\right] \quad (s5\text{-}6)$$

And so

$$r_{\rm rec}(V_{\rm d}) = r_{\rm rec,0} \exp\left(\sqrt{\frac{q r_{\rm C} c_2}{kT}(V_{\rm d})^{\beta}}\right)\left[1 - \psi^2 \frac{q r_{\rm C} c_2}{kT}(V_{\rm d})^{\beta}\right] \quad (s5\text{-}7)$$

Apparently $r_{\rm rec,0}$ is proportional to carrier mobility near the interface $\mu_{\rm int}$ and the number of chargeable sites N_0, but decays fast as function of the Schottky barrier height φ_B. The parameter $r_{\rm rec,0}$ is independent of V_d and well characterize the interfacial injection conditions with surface recombination process. For calculations in Figure 8, the used parameters are in Table S2. The Figure 8e is drawn by plotting $r_{\rm rec}$ against $R_{\rm c,int}$ (by Eq. S6-2) for different V_d and $\varphi_{\rm eff}$.

Table S2. Parameters for simulation shown in **Figure 8**.

C1	α	C2	β	C3	μ(cm²/Vs)	γ
0.01	1	2e7	1	1	0.1	1
**N_0(cm^{-3})**	**$\varphi_{\rm eff}$(eV)**	**r_c(m)**	**T(K)**	**ε**	**S**	
1e6	0.1~0.3	1.6e-8	300	3.5	1	

(3) Note that $r_{\rm rec}$ here is an interface property and is different from the original "*Langevin's rate of recombination*" in the bi-polar bulk OSC, which is defined by the total number of electron-hole recombination events per unit time (in one second) and per unit volume (in one cm^3) and is also proportional to mobility.

Part 6. Derivation of $R_{c,int}$ and Equation 16 in the main text

The voltage across the interface V_a and local electric field ϵ at the interface and are affected by V_d. Assume the former is $V_a = c_1(V_d)^{\alpha}$ and the latter is $\epsilon = c_2(V_d)^{\beta}$, and charge mobility near the interface follows the field-effect mobility $\mu_{int} = c_3\mu^{\gamma}$. Yet because the change in c_1 (characterizing the interfacial voltage) and c_2 (characterizing the interfacial field) simultaneously decrease or increase with changing V_d, the parameter c_1/c_2 slowly changes with V_d, and so can be approximately considered as constant. According to the model proposed by *Scott* et. al, the net injected current is:

$$J = 4\psi^2 N_0 q k_3 \mu^{\gamma} c_2 (V_d)^{\beta} \exp(-\varphi_{eff}/kT) \exp\left[\sqrt{\frac{er_C}{kT} c_2 (V_d)^{\beta}}\right] \quad \text{(s6-1)}$$

Then we have the interfacial contact resistance $R_{c,int}$ as,

$$R_{c,int} = \frac{V_{int}}{JS} = \frac{c_1}{c_2 c_3 S}(V_d)^{\alpha-\beta} \times \frac{1}{4\psi^2 N_0} \times \frac{1}{q\mu^{\gamma}} \times \exp(\varphi_{eff}/kT) \times \exp\left[-\sqrt{\frac{qr_C c_2}{kT}(V_d)^{\beta}}\right] \quad \text{(s6-2)}$$

Here S is the contact area. Let $\psi = (\psi_{max} - \Delta\psi)$, the first term is the maximum value of ψ for different V_d in the investigated region and the second term is a function of V_d. Because ψ is a weak function of ϵ, so $\Delta\psi \ll \psi_{max}$. Let $B = \sqrt{\frac{qr_C c_2}{kT}}$ and assume $\alpha = \beta = 1$, and then we use the Talyor expansions,

$$R_{c,int} = \frac{c_1}{4c_2 c_3 N_0 q\mu^{\gamma} S} \exp\left(\frac{\varphi_{eff}}{kT}\right) \exp\left(-B\sqrt{V_d}\right) \times \frac{1}{\psi_{max}^{\;2}} \left(\frac{1}{1-\frac{\Delta\psi}{\psi_{max}}}\right)^2 \cong$$

$$\frac{c_1}{4c_2 c_3 N_0 q\mu^{\gamma} S} \exp\left(\frac{\varphi_{eff}}{kT}\right) \exp\left(-B\sqrt{V_d}\right) \frac{1}{\psi_{max}^{\;2}} + \frac{c_1}{4c_2 c_3 N_0 q\mu^{\gamma} S} \exp\left(\frac{\varphi_{eff}}{kT}\right) \exp\left(-B\sqrt{V_d}\right) \frac{2\Delta\psi}{\psi_{max}^{\;3}} \quad \text{(s6-3)}$$

We can simplify it as,

$$R_{c,int} \cong A\exp\left(-B\sqrt{V_d}\right) + R_0 \quad \text{(s6-4)}$$

$$A = \frac{c_1}{4c_2 c_3 N_0 q\mu^{\gamma} S} \frac{1}{\psi_{max}^{\;2}} \exp\left(\frac{\varphi_{eff}}{kT}\right) \quad \text{(s6-5)}$$

$$R_0 = \frac{c_1}{4c_2 c_3 N_0 q\mu^{\gamma} S} \frac{2\Delta\psi}{\psi_{max}^{\;3}} \exp\left(-\alpha\sqrt{V_d}\right) \exp\left(\frac{\varphi_{eff}}{kT}\right) \quad \text{(s6-6)}$$

As $R_0 << R_1$ at small V_d, R_0 can be regarded as a constant of V_d when discussing the total $R_{c,int}$ at small V_d.

Moreover, by combining Eq. (s6-2), (s5-4), and (s5-5),

$$R_{\mathrm{c,int}} = \frac{1}{r_{\mathrm{rec}}} \times \left[\frac{c_1}{c_2 c_3 S}(V_{\mathrm{d}})^{\alpha-\beta} \frac{1}{4\psi^2} \frac{q^3}{16\pi\varepsilon\varepsilon_0 (kT)^2} \frac{1}{1-\psi^2 \frac{q r_{\mathrm{C}} c_2}{kT}(V_{\mathrm{d}})^{\beta}}\right] \tag{s6-7}$$

If the term in the bracket is a weak function of V_{d}, we have $R_{\mathrm{c,int}} \propto \frac{1}{r_{\mathrm{rec}}}$.

References

[1] M. Marinkovic, D. Belaineh, V. Wagner, D. Knipp, *Adv. Mater.* **2012**, *24*, 4005.
[2] C. Liu, Y. Xu, G. Ghibaudo, X. Lu, T. Minari, Y.-Y. Noh, *Appl. Phys. Lett.* **2014**, *104*, 013301.
[3] S. W. Luan, G. W. Neudeck, *J. Appl. Phys.* **1992**, *72*, 766.
[4] J. C. Scott, G. G. Malliaras, *Chem. Phys. Lett.* **1999**, *299*, 115.
